# The Impact of the COVID-19 Pandemic on Domestic Abuse Against Turkish Immigrant Women in Germany

**DOI:** 10.1192/j.eurpsy.2023.1662

**Published:** 2023-07-19

**Authors:** D. E. D. Cindik-Herbrüggen

**Affiliations:** Psychiatry, Neuro-Psychiatrisches Zentrum Riem, Munich, Germany

## Abstract

**Introduction:**

The most common but most hidden form of violence against women is domestic violence. One out of every three women in the world is exposed to physical, psychological or sexual violence by her close partner at any time in her life (Ünal and Gülseren, 2020). As a result of the social isolation measures and quarantine regulations brought by the Covid-19 pandemic, reports of domestic violence against women have increased.

**Objectives:**

This study aimed to investigate the relationship between psychological violence, psychological maltreatment and depression, anxiety among Turkish immigrant women living in Germany during the COVID-19 pandemic.

**Methods:**

The Profile of Psychological Abuse of Women, Psychological Maltreatment of Women Inventory, Generalized Anxiety Disorder-7 (GAD-7) Scale, and The Patient Health Questionnaire-9 (PHQ-9) were delivered to participants.

**Results:**

Our results show that participants who had been exposed to psychological abuse and domestic violence reported to have higher depression and anxiety scores. Furthermore, participants with low income and married to spouses with no formal education or only primary school graduates were reported to have higher depression, anxiety, domestic abuse and psychological maltreatment scores during the COVID-19 outbreak.

**Conclusions:**

Our findings demonstrated that Turkish immigrant women who had experienced psychological abuse and domestic violence by their partners during the pandemic were reported to have higher depression and anxiety.

**Disclosure of Interest:**

None Declared

